# Cholera Infantum

**Published:** 1887-07

**Authors:** James B. Baird

**Affiliations:** Atlanta, Ga.


### CHOLERA INFANTUM.*

BY JAMES B. BAIRD, M. D., ATLANTA.

When we reflect that about one-half of all the deaths in this community occur among children under five years of age, and that about one-fifth of all recorded deaths are ascribed to diarrhœal diseases, the practical importance to the active practitioner of the subject selected for discussion this evening is at once manifest.

The looseness with which the term cholera infantum has been used, and the rarity of disordered conditions it has been made to cover, create considerable confusion in any attempt to deal with the subject; for, to some minds, it suggests one series of morbid phenomena, while to others it calls up a picture presenting very different and possibly much darker hues. The signification of the term ranges in its elastic accommodation from the mild summer diarrhœa of infancy and early childhood to those

*Remarks made before the Atlanta Society of Medicine in opening the regular discussion May 3, 1887.

dreadful choleraic disturbances that burst with all the suddenness and fury of a terrific thunder-storm upon the tranquil happiness of a peaceful household, and which sometimes relentlessly terminate, in a few hours, the life of the innocent and unsuspecting little victim.

The familiar synonyms for this term are gastro-enteritis choleraformis, gastro-enteric catarrh, follicular enteritis of infants, choleric fever of children, choleraic diarrhœa, sporadic infantile cholera, summer cholera, summer complaint, weaning brash, watery gripes, etc. Properly, the term applies to cases of "acute gastro-intestinal catarrh in young children, characterized by large watery stools, profuse vomiting, high temperature, great prostration and rapid wasting." It has been proposed to include, however, all those affections of infants in the first or second year, especially at the time of dentition, occurring in towns, or populous rural districts, in hot weather, attended by vomiting during a portion, but not necessarily the whole course of the attack, with diarrhœa, acute or chronic, usually producing emaciation and threatening, if not actually causing, fatal debility. For the purposes of this discussion, it is convenient to employ the term in its conventional and widest sense, as it is my desire to invite your attention mainly to the causes, prevention and treatment, and as these are common to a variety of gastro-intestinal disorders, it is practically important that the most comprehensive interpretation should be given to the general and representative term, which, with this explanation, it is understood, stands for several affections.

Our mortuary lists show an enormous increase in infantile mortality from diarrhœal diseases during the spring, summer and autumn months. In this latitude, from May to November, most marked in mid-summer, as compared with the remainder of the year. These deaths are recorded as cholera infantum, dysentery, diarrhœa, entero-collitis, gastro-enteritis, gastro-duodenitis, summer complaint, inanition, marasmus, teething, convulsions, etc. Thus, while the names employed by those who sign death-certificates are descriptive of the most prominent features, or the most violent symptoms, there is a general resemblance in the essential phenomena of the several disorders, which reveals relationship, and indicates that these individual members all belong to the same family, and while, of course, they are not identical, speaking generally, they are all controlled by the same general laws, influenced by the same general causes and amenable to the same general treatment.

It may be stated at the outset that this disease, or, as has been shown, these diseases are encountered, as a rule, within the first two years of life and in the warmer seasons of the year. They are much more common in cities than in the country; they are more frequent in hand-fed children than in those naturally nourished. Hence we observe that the question of etiology must relate to age, season, environment and food.

Age, of course, is an essential element in causation. The first half year is perhaps most prolific of these derangements, and after two years they are comparatively rare. The "second summer" is probably most dreaded in popular estimation.

Heat has been mentioned as an active factor in the production of cholera infantum. The influence of a high atmospheric temperature may be excited directly upon the subject by lowering the tone of the system, by lessening the power of resistance and by exciting an increased nervous irritability. We see the direct effects of heat in an extreme degree in cases of sunstroke; should we then not expect to find the sensitive organization of a delicate infant impressed by this agency when it is exposed to its influence? Heat may also act indirectly by calling into activity various terrene agencies which may serve to develop a latent predisposition or to increase the susceptibility to local irritants, as undigested food or impure water.

Food not adapted to the digestive capacity of the child is a palpable and an immediate cause of many and very severe attacks. In connection with age and heat, this is probably by far the most frequent exciting cause of these disorders.

Bad sanitary conditions and surroundings, as crowded habitations, defective ventilation, uncleanliness, contaminated water, an impure atmosphere, no out-of-door life, in short, the conditions that obtain in tenement houses in large cities, contribute to the predisposition as well as to the severity and fatality of diseases of this character.

The fact should be appreciated that a combination of several of these causes is usually necessary for the production of cholera infantum. They rarely operate singly. Moreover, the fact that these several causes attain greater activity during the period of dentition is due, beyond a doubt, in my mind, to the process of teething itself. It is true this is a physiological process, but it may and often does develop pathological conditions. Besides the impress made on the nervous system, the gums may become red, hot and painful. The child is rendered irritable and fretful; sleep may be disturbed or broken and unrefreshing. We have a state akin to that produced by an abscess, inflamed hemorrhoids or acute tonsilitis. The reflex excitation is worthy of note. Let us remember the marvelous possibilities of these influences. The striking nervous phenomena associated with certain uterine or gastric disorders, or with urethral stricture, may serve as illustrations. I most decidedly do not concur in the opinion expressed by a recent writer* to the effect that "there is no ground for the belief that dentition is a factor in its causation, save that the great developmental and functional activity of the intestinal follicles during this period is a powerful predisposing cause to all kinds of intestinal derangement." Surely the relief which sometimes follows a simple incision of an inflamed gum may be admitted as evidence in estimating the influence of even one little tooth as the starting point of a long train of threatening symptoms. Nevertheless, it is unquestionably true that the imperfect development of the digestive organs at this time of life exposes them to serious disturbance in consequence of the ingestion of unphysiological and, for the child, indigestible food.

It has been supposed that this disease was peculiar to this country, but it is described both by British and by continental

*W. J. Conklin in Wood's Reference Handbook of the Medical Sciences, Art. Cholera Infantum, Vol. III.

writers, yet it certainly prevails to a much greater extent in America than it does abroad, and is pre-eminently an American disease. In view of the influence of heat in its causation, it would seem reasonable to infer that it would be more frequent and more fatal in our Southern States, where the summers are longer and the average temperature higher than in more northern latitudes; but Austin Flint is authority for the assertion that the disease is more common in the Northern and Middle States than in the Southern States.

The morbid anatomy is, of course, variable, for we are now dealing with several diseases, but the changes, even in the more typical forms of cholera infantum, are not constant. The mucous membrane of the digestive tract may be unnaturally pale, exsanguined, or it may be uniformly congested and softened, or reddened only in part. Peyer's patches and the solitary glands are sometimes enlarged and project above the level of the bowel. These may soften, and breaking down form little ulcers grouped over the surface of the intestine. The mesenteric glands may be found congested. The brain is anæmic and, like other parts of the body, becomes shrunken and wasted; serum is effused into the ventricles and under the meninges; usually there is pulmonary hypostatic congestion. No important lesion has been observed in the liver, spleen, lungs or kidneys.

The onset of an attack may be sudden or it may occur intercurrently to mild diarrhœa. It is caused sometimes, under these circumstances, by a single dietary indiscretion. Frequent watery vomiting and purging constitute the prominent features of cholera infantum. If these symptoms persist, disorganization of the blood, speedy wasting and rapid exhaustion follow. Moderate fever of a remittent type is usually present. To the touch the surface may appear unnaturally cool, while the thermometer may at the same time indicate a temperature of 101 to 105 or even 108 degrees. The mouth is dry, the lips are fissured, the features are pinched and withered. Some cases resemble closely the sporadic cholera of adults.

The duration of a severe attack may be from six or eight hours only to several days. In fatal cases collapse usually follows the violent symptoms in one to three days. If convalescence ensues the attack may eventuate in a chronic state marked by diarrhœa and occasional vomiting. On the other hand, if the progress is unfavorable, the urine is greatly diminished, as we should expect in view of the abundant watery discharges. It is sometimes suppressed. Drowsiness lapses into coma; convulsions frequently occur and death soon follows.

The prevention of cholera infantum is so intimately associated with what has already been said under the head of causation, and with what remains to be said in connection with treatment, it is scarcely necessary to refer to it otherwise, except for the purpose of impressing the indispensable importance of observing a proper infantile hygiene. This means a great deal, and demands our best efforts, and is worthy of our constant care. By so doing we may not only avoid immediate trouble, but forestall at the same time remote dyspeptic disorders, which, beyond a doubt, frequently have their origin in the dietetic imprudences of the first few years of life.

The diagnosis is sufficiently easy, and by attention to the prominent symptoms it may be made with confidence.

The question of a possible tuberculous diarrhœa merits serious consideration in subjects over eighteen months old. It is said to be of rare occurrence under this age.

The prognosis, of course, must be based upon the condition found in each individual case. In these diseases, however, hope of ultimate recovery need not be abandoned under the most discouraging assemblage of symptoms. The affections do not always involve serious structural lesions, but the threatening issue may be due solely to innutrition and exhaustion. So, if the patient can only be kept alive, recovery may finally be reached.

In the treatment of these disorders the question of food is of prime and insurmountable importance. Human milk is nature's food for infants. Upon their natural nourishment they thrive; deprived of it they languish and thousands die annually from this cause alone. When possible, then, the mother should nourish her child. There can be, I apprehend, no disputation on this point. If the supply of milk is deficient or defective, it may sometimes be increased and improved by a proper hygiene and diet on the part of the woman. It is extremely desirable to accomplish this result. If success in this direction should prove to be beyond reach, or if from debility, disease or the existence of some constitutional taint it is inexpedient for the mother to nurse her baby, a wet-nurse, if possible, should be secured. Sometimes, however, children will not take the breast of a strange woman, especially if they have been weaned and when they are sick. Hence we may be driven to the necessity of prescribing a substitute for human milk. What should this be ? is the vital question. Undoubtedly the most available, and practically the best substitute, is cow's milk properly prepared. Milk of good quality is indispensable. It should be obtained from a cow in good order, suitably stabled and fed, and having calved within six months.* The feed of the cow should be restricted to bran, meal and hay, with an abundance of salt and of pure water. Quality, not quantity, is sought, for other management will cause a larger yield of milk. I have known children who could not digest the milk of Jersey cows, but who thrived on the milk of common stock belonging to the same herd. I do not offer any explanation of this fact. It seems, moreover, to be a rare exception to the rule.

The milk, carefully selected, should be methodically diluted to suit, not the age in months, but the development and digestive strength of the individual child. It should always be remembered that it is the food which is digested and appropriated which nourishes and sustains the body. This is not always in proportion to the amount ingested. The quantity taken into the stomach may, under certain circumstances, be large—excessive, yet literal starvation may ensue.

The following table will serve as an approximative guide to the requisite degree of dilution by measure—subject, of course, to the modification just referred to, namely, the development of the child and the state of its digestive organs:

---

*I am informed that in some parts of Europe cows are spayed for the purpose of improving the quality of the milk and of making the supply continuous.

First Week—Milk, 1; water, 3.
First Month—Milk, 1; water, 2.
Third Month—Milk, 1; water 1½.
Fifth Month—Milk, 1; water, 1.
Eighth Month—Milk, 2; water, 1½.
Twelfth to Eighteenth Month—Milk, 2; water, 1.

This may be accepted as a fair standard of dilution in health. When the digestion is weakened by disease, the dilution must be proportionately increased.

The upper half only from double the amount of milk required should be used. From this portion the quantity needed for a single meal should be dipped up after the milk has stood for three or four hours—in warm weather on ice or immersed in cool water—in order that sufficient cream may be procured to compensate for the addition of the water. A little white sugar must also be supplied to compensate for the dilution. The mixture ought then to be warmed by immersing the vessel containing it in hot water for a few moments, and it should be given by preference out of a nursing-bottle. When the dejections contain curd-like masses, further dilution is required; occasionally the addition of a little lime water is serviceable.

Condensed milk is not a trustworthy substitute for the fresh article. It contains an excessive proportion of sugar. The equalizing of the several constituents, and its ready reduction to the standard of human milk, cannot be intelligently accomplished as directed above. I know of no way to test its quality before it is taken. Some children seem to thrive upon it, at least for a time, but I cannot indorse its general use Occasionally it may prove a convenient and temporary resource.

It is not necessary or desirable to boil cow's milk; with proper care no objectionable changes in it need be feared. It has appeared to me that the minute coagula which cause the slight thickening of boiled milk must render it more difficult of digestion.

I have been favorably impressed by the proposition to peptonize the milk. By this process, the coagula, when the milk is taken into the stomach, are rendered more flocculent and less firm. Theoretically, the proposition is sound. As yet, my experience with it does not enable me to express a positive opinion as to its practical utility. If this "artificial digestion" should be carried too far, it imparts a bitter taste to the milk, which is, of course, offensive to the child.

Starchy foods are insufficient for nourishment and are otherwise harmful. These and the whole list of proprietary and commercial infant's foods should be proscribed—not that all of these preparations are equally injurious, but none are essential, many are seriously objectionable, and it is consequently a safe rule to discard all—every one. I emphasize this advice in the interest of helpless infancy.

Care should be taken not to over-feed the subject of intestinal disorders in our anxiety to sustain the strength. Sometimes it is desirable to withhold all food for several hours. While it is frequently necessary to give food at short intervals, at no time should it be given continuously. The physiological rest of the stomach is required. Incessant activity should not be provoked. Give water or ice frequently, but not too much at one time. Water requires no digestion and does not tax the stomach, but the mere bulk from excessive ingestion or from considerable accumulation in the stomach may cause vomiting. Food, taken in very small quantity, does not cause peritalsis. Sometimes chicken-broth, frozen beef-tea, diluted beef essence, raw beef or mutton reduced to a pulp or the rectal injections of meat juice are serviceable. The tranquillizing effect of a warm or tepid bath should not be overlooked. In excessive heat the cold bath is commended. Immerse the child in water at one hundred degrees, gradually reduced to eighty or seventy degrees. Continue the immersion, with a cold cloth on the head, for eight or ten minutes. The same procedure may be repeated, if the results are satisfactory, two or three times daily. If depression follows, or symptoms of failure or collapse ensue, this treatment should be promptly abandoned. The utmost deliberation and gentleness is needful to avoid giving alarm to the little patient. Salt may be added to the bath. In the case of timid children, sponging may be substituted. Alcohol sponging is often very grateful. Enemata of cool or cold water may rarely be appropriate for accomplishing a reduction of the temperature. Enemata of salt-water are serviceable for allaying troublesome tenesmus.

Good nursing is of incalculable value. The room should be large, airy, well ventilated, agreeably shaded and quiet. Loud talking, or conversing even in subdued tones, should be interdicted. The utmost cleanliness should be observed in the bedding, clothing, nursing-bottle, etc. A change of residence is frequently the promptest and most potent means of cure that can possibly be applied, especially from the city to the country, to the sea-shore or to the mountains. The beneficial effect of change, even in apparently hopeless cases, is sometimes almost magical.

In the selection of drugs appropriate to any case in which the above suggestions as to diet and hygiene do not suffice to secure relief, we must bear in mind that we may have to deal under this general term with sporadic cholera, diarrhœa from indigestion, enteritis, gastro-enteritis, entero-collitis or colo-enteritis and dysentery. I shall ask you, gentlemen, in discussing the applicability of drugs, to bear this fact in mind, and to remember that a discriminating judgment must be exercised in selecting the most suitable remedies for a given case from those herein enumerated in order to best meet the indications presented by these several conditions. Among the digestive tonics pepsine and bismuth come, in my estimation, first. Let the quality of the pepsine be good, and give it in full doses. I prefer the pure—that is, not the saccharated pepsine. I am in the habit of ordering equal parts of pure non-hygroscopic pepsine, subnitrate of bismuth and white sugar, of each from one to three grains or more, according to age, to be taken dry on the tongue three or four times, or oftener, daily.

Opium is sometimes indispensable as a temporary assistant, though great circumspection should be observed in its use. It may be given as morphine, the deodorized tincture or paregoric.

The hypodermatic use of drugs is not free from danger in infants. The risk of tetanus should not be overlooked. I infinitely prefer suppositories or rectal injections. Suppositories of opium, or of the watery extract, may be combined with extract of belladonna or extract of hyoscyamus and acetate of lead. Laudanum injections are a valuable resource. To these may be added, if deemed advisable, sulphate of copper, acetate of lead and nitrate of silver. If the latter drug is employed the injection should be preceded by bathing the arms and adjacent portion of the rectum with salt-water to avoid distressing tenesmus, which it is liable to produce. Dovers powder is an eligible form for the exhibition of opium.

One or two drops of the deodorized tincture of opium on a lump of sugar have often proved, in my hands, a convenient mode of administration. Tincture of opium may be combined with tincture of krameria and chalk mixture—the elixir of orange being an elegant vehicle. Perhaps some of the natural mineral water may prove, in exceptional cases, available. Chloral, to procure sleep and to calm nervous excitement, is a valuable remedy. A dose of castor oil, or possibly small doses of calomel, are indicated in some cases. The latter may be combined with Dover's powder, not for its purgative effect, but to improve the secretions. This, gentlemen, may sound like old-time doctrine, but it has a practical meaning. I sometimes fear that we go too fast; that in our eager haste to find and to follow something new we surrender the substance for the shadow, and that in our headlong pursuit of scientific theories we sadly miss some of the substantial results achieved by our venerated fathers in medicine, guided as they were by their hard common sense.

Aromatic sulphuric acid, muriatic acid, carbonate of ammonia, chloroform, hydrocyanic acid, camphor, Fowler's solution, tincture of nux vomica in small doses, and carbolic acid, all enjoy certain repute, and are confidently indorsed by respectable authorities. The antiseptic method of treatment bids fair to become fashionable. Creosote, in one-eighth to one-sixth drop doses in mucilage, after vomiting, is said to exert sometimes a magical influence. Quinine, of course—quinine heroically, to reduce the temperature and to kill the terrible germs, the bacilli that are supposed to swarm in the blood of every one who has an ailment, from a corn on his toe to a cancer on his nose. I do not approve of this abuse of a valuable remedy. Brandy is very important, not only to sustain strength for the moment, but to ward off asthenia. We must at times foresee this evil and prepare to meet it. I have not formed a favorable opinion of port wine, and I think the wine of pepsine an illogical preparation. I never prescribe it.

Externally counter-irritation, with mustard, turpentine, spiced poultices, camphorated oil and hot cloths, fulfill clearly-defined indications.

Now, gentlemen, while I have given you a long list of so-called remedies, let me earnestly counsel you not to be officious in the use of drugs. Reliance upon them sometimes contributes to your defeat in those cases, and they are legion, where indigestion is the prime underlying vice. Look to the cause of the attack and seek to correct it. Avoid complicated prescriptions. Eschew highly-flavored and syrupy compounds. Strive to do no harm, even if you fail to do good. Let your zeal be tempered with prudence, and, as the fatal season is at hand, may you be prospered in your work.

